# Anti-Inflammatory Activity of AF-13, an Antioxidant Compound Isolated from the Polar Fraction of *Allomyrina dichotoma* Larva, in Palmitate-Induced INS-1 Cells

**DOI:** 10.3390/life11060470

**Published:** 2021-05-24

**Authors:** Kyong Kim, Eun-Young Park, Dong-Jae Baek, Chul-Young Kim, Yoon-Sin Oh

**Affiliations:** 1 Department of Food Nutrition, Eulji University, Seongnam 13135, Korea; kim_kyong@hanmail.net; 2 Natural Medicine Research Institute, College of Pharmacy, Mokpo National University, Jeonnam 58628, Korea; parkey@mokpo.ac.kr (E.-Y.P.); dbaek@mokpo.ac.kr (D.-J.B.); 3 College of Pharmacy, Hanyang University, Ansan 15588, Korea; chulykim@hanyang.ac.kr

**Keywords:** *Allomyrina dichotoma* larva, AF-13 fraction, palmitate, INS-1, inflammation, apoptosis

## Abstract

This study was conducted to evaluate the fractions isolated from *Allomyrina dichotoma* larva extract (ADLE) that exhibited anti-apoptotic and anti-inflammatory effects. A total of 13 fractions were eluted from ADLE by centrifugal chromatography (CPC), and the polar AF-13 fraction was selected, which exerted a relatively protective effect against fat-induced toxicity in INS-1 cells. AF-13 treatment of palmitate-treated INS-1 cells decreased the expression level of apoptosis-related proteins and DNA fragmentation. AF-13 also significantly inhibited the production of nitric oxide and reactive oxygen species and the triglyceride content induced by palmitate, and the effect was found to be similar to that with ADLE treatment. Palmitate upregulated the expression of cyclooxygenase-2 (COX-2) and inducible nitric oxide synthase (iNOS) through the activation of NF-κB p65; however, this effect was significantly attenuated by AF-13 treatment. In conclusion, AF-13 is one of the major components of ADLE responsible for anti-apoptotic and anti-inflammatory activities.

## 1. Introduction

Inflammation causes insulin resistance, the primary trigger of type 2 diabetes, and may also be a major cause of fatal diabetes-related complications. Continuous exposure to saturated free fatty acids contributes to β-cell dysfunction caused by lipotoxicity and inflammation associated with several deleterious effects on physiological systems [1]. 

Palmitate triggers a typical event, i.e., the induction of nitric oxide synthase (iNOS), an enzyme that catalyzes the intracellular generation of the cytotoxic free radical NO. Nuclear factor-κB (NF-κB), which is activated by inflammatory responses during viral and bacterial infections, is involved in the expression of *iNOS* genes [2]. Moreover, oxidative stress due to an increase in intracellular reactive oxygen species (ROS) levels plays a central role in insulin resistance and pancreatic β-cell death during the progressive deterioration in glucose tolerance and development of type 2 diabetes [3,4,5,6].

Living organisms have their own natural antioxidants and substances that defend against ROS [7]. In particular, insects possess antioxidant defense systems that contain antioxidant enzymes such as superoxide dismutase, catalase, glutathione transferase, and glutathione reductase, which can suppress the disproportions caused by excessive free radical production [8,9]. The horn beetle *Allomyrina dichotoma* has been used in various folk remedies in several countries and is believed to contain useful biologically active substances.

In South Korea, the beetle was authorized for use in food and feed in 2010, and the recently known pharmacological properties include anti-liver fibrosis, antineoplastic, anti-obesity, anti-Alzheimer’s, antioxidant, and antidiabetic effects, which have been primarily reported in the beetle larva [10].

We recently showed that *A. dichotoma* larvae (ADL) alleviated hepatic insulin resistance in high-fat-diet-induced diabetic mice [11] and prevented free fatty acid (FFA)-induced lipotoxicity in pancreatic beta cells [12]. We also reported that it attenuated intestinal barrier disruption by altering inflammatory response and tight junction proteins in lipopolysaccharide-induced Caco-2 cells [13].

Although the nutritional constituents of ADL have been recognized as an alternative food source of high-quality protein that may be lacking in the future, there is a scarcity of knowledge on the physiological activities of the primary active ingredients of ADL. Therefore, this study was designed to investigate the major components of ADLE. Moreover, we examined whether AF-13 reduced lipotoxicity in palmitate-induced INS-1 cells and the effect were compared with the crude ADLE.

## 2. Materials and Methods

### 2.1. Fractionation of ADLE by Centrifugal Partition Chromatography

*A. dichotoma* larvae (ADL) grown for about 100-120 days from eggs (the third larvae stage) were purchased from Yechun Bugs land (Yecheon-gun, Gyungsangbuk-do, Korea). Crude ADL extract (ADLE) was prepared as described previously [11].

Fractionation of ADLE was performed by centrifugal partition chromatography (CPC) using a combination of an SCPC-1000 (Armen Instrument, St-Ave, France) apparatus and a Spot Prep II HPLC instrument. The CPC was operated with a gradient solvent system composed of *n*-hexane–acetonitrile–water, ethyl acetate–acetonitrile–water, and water-saturated *n*-butanol–acetonitrile–water (each 10:2:8, *v*/*v*/*v*). The lower phase of *n*-hexane–acetonitrile–water was used as the stationary phase, and its upper phase, as well as ethyl acetate–acetonitrile–water, and water-saturated *n*-butanol–acetonitrile–water were eluted with a flow rate of 10 mL/min for 0–30 min (100% A), 30–90 min (100% A–100% B), 90–210 min (100% B–100% C), and 210–310 min (100% C). Then, all of the remaining samples were washed off with methanol elution. The effluent was monitored at 270 nm in the scan mode (254–400 nm). ADLE (5.0 g) was dissolved in 20 mL of mixed upper and lower phases and subjected to the CPC system.

### 2.2. HPLC Analysis

Samples were analyzed using a Waters Alliance 2695 HPLC system coupled with a Waters Micromass ZQ detector (Waters, Milford, MA, USA) with an Inno C18 column (2.0 × 150 mm, 5 μm, Youngjin Biochrome, Seongnam, South Korea). The mobile phase was composed of acetonitrile containing 0.1% formic acid (A) and water containing 0.1% formic acid (B). The gradient elution conditions were as follows: 0–20 min, 5–50% A; 20–40 min, 50–100% A; and 40–50 min, 100% A. The flow rate was 0.3 mL/min, and the injection volume was 10 μL. The micromass ZQ mass spectrometer equipped with an electrospray ionization source (ESI) probe working at 120 °C was operated in the positive ion mode and in the negative ion mode. Nitrogen was used as desolvation gas at a flow rate of 900 L h^−1^. The desolvation temperature was 350 °C. Mass values of 200–1200 u were measured. Capillary and cone voltages were 3500 V and 60 V for ESI^+^, and 3000 and 45 V for ESI^−^, respectively. Data acquisition and processing were performed using MassLynx 4.1.

### 2.3. Total Phenolic Content and Antioxidant Activity

Total phenolic content was measured using the Folin–Ciocalteu reagent (Sigma, St. Louis, MO, USA). A standard curve was also plotted using gallic acid. Results are expressed as milligrams per gram of fraction of gallic acid equivalent (GAE). Antioxidant activity was evaluated using the DPPH assay with a synthetic radical, 2,2-diphenyl-1-picrylhydrazyl, according to the method reported by Paduch et al. [14]. Radical scavenging activity was calculated as a percentage of DPPH decolorization compared with the control. Antioxidant ability of the sample was expressed as IC_50_ values.

### 2.4. Cell Culture, Palmitate Treatment, and Cell Viability

INS-1, known as a murine pancreatic β-cell line, was sub-cultured in RPMI 1640 medium (Paisley, Gibco, UK) containing 1% penicillin/streptomycin (Welgene, Daegu, Korea) and 10% fetal bovine serum (Gibco) in an atmosphere of 5% CO_2_ at 37 °C. To facilitate the absorption of saturated fatty acids into cells, palmitic acid (PA, Sigma) was prepared by dissolving 20 mM PA in 10 mM NaOH at 70 °C for 30 min, and then conjugated with 5% BSA solution dissolved in DPBS at a ratio of 1:3. After seeding at 2.5 × 10^4^ cells/well of INS-1 cells for overnight, the cells treated with 0.4 mM palmitic acid with or without 500 µg/mL ADLE or 25, 50, and 100 µg/mL fractions for 24 h. Cell viability was assayed by MTT method (Duchefa Biochemie BV, Haarlem, The Netherlands). At the end of the reaction, after removing the supernatant, MTT at 1mg/mL was added to well by 100 μL each, and the cells were incubated for 2 h. Once completed, purple insoluble formazan crystals were dissolved by 200 μL of 2-propanol and measured at 540 nm (TECAN Group Ltd., Shanghai, China).

### 2.5. Measurement of Nitrite, ROS, and ATP Levels

INS-1 cells were seeded at a density of 2.5 × 10^4^ cells/well on a 96 opaque black-well plates for overnight. The cells treated with 0.4 mM palmitic acid with or without 500 μg/mL ADLE and 100 μg/mL AF-13 for 24 h. After completion of the reaction, the supernatant reflecting NO generation in extracellular was collected and was reacted 1:1 with Griess reagent (1% sulfanilamide/0.1% N-(1-naphthyl)-ethylenediamine dihydrochloride/2.5% H_3_PO_4_) at room temperature for 10 min. To decide the nitrite concentration, sodium nitrite was used as a standard and measured at an absorbance of 540 nm. Intracellular ROS generation from reacted cells was evaluated using an oxidative-sensitive 2′,7′-dichlorodihydrofluorescein diacetate (DCFH-DA; molecular probe) fluorescent probe. Briefly, cells were washed twice with DPBS, incubated in the dark with 10 μM DCFH-DA for 30 min. After the reaction, cells were washed three times with DPBS and 100 μL of DPBS was added to each well. The fluorescence values of dichlorofluorescein (DCF) in the cells was detected using a fluorescence spectrophotometer at excitation and emission wavelengths of 488 and 535 nm, respectively. Cellular ATP levels were measured based on luminescence using the Perkin-Elmer ATPlite system according to the manufacturer’s instructions. Cells were washed twice with DPBS after reaction under the same conditions as above. In brief, the provided mammalian cell lysis solution, which releases adenine nucleotides from cells and inactivates endogenous ATP degrading enzymes, was primary treated, followed by addition of luciferase and D-luciferin to react with the producing ATP.

### 2.6. Cellular Lipids Analysis

INS-1 cells were seeded at a density of 1.0 × 10^6^ cells/well on a 6 well plates for overnight. The cells treated with 0.4 mM palmitic acid with or without 500 μg/mL ADLE and 100 μg/mL AF-13 for 24 h. The cells were washed twice with DPBS and collected by cell scraper. The cells were extracted with methanol:chloroform (1:2, *v*/*v*) shaking horizontally for 2 h, and centrifuged at 2500× *g* for 10 min. The organic phase was collected, evaporated under nitrogen, and resuspended in Triton X-100:ethanol mixture (1:1, *v*/*v*). Cellular lipid content was determined by using the triglyceride (TG-S) reagent kit (Asan Pharmaceutical Co., Seoul, Korea). Data were normalized for differences in protein concentration in the cell extracts.

### 2.7. DNA Fragmentation

Cytoplasmic histone-associated DNA fragments in palmitate-induced cell death were analyzed using the Cell Death Detection enzyme-linked immunosorbent assay (ELISA) Plus kit (Roche Molecular Biochemicals, Mannheim, Germany) for the relative quantification of apoptotic cells. The extraction of the cytoplasmic fraction was carried out according to the manufacturer’s protocol.

### 2.8. Preparation of Nuclear Extracts and Western Blotting

Nuclear protein was isolated from INS-1 cells using NE-PER nuclear and cytoplasmic extraction reagents (Thermo Fisher Scientific, Waltham, MA, USA) and evaluated using the NF-κB pathway sampler kit (Cell Signaling Technology, Danvers, MA, USA).

Western blotting was conducted as described previously [11]. Briefly, 10–50 mg of proteins was separated by 10% SDS–PAGE, transferred to nitrocellulose blotting membranes, and incubated with primary antibodies. After washing the membranes with TBST, they were incubated with secondary antibodies. Protein bands were detected using an ELC kit (Millipore, Burlington, MA, USA). Bands were normalized to β-actin and quantified using the Quantity 1 version 4.6.7 software (Bio-Rad, Hercules, CA, USA).

### 2.9. Statistical Analysis

Statistical analysis was performed using the SPSS 20.0 software (IBM SPSS ver. 20.0.0 for Windows; IBM Co., Armonk, NY, USA). Results are expressed as mean ± standard deviation. Significance of differences among groups was analyzed by LSD comparisons tests. Statistical significance was set at *p* < 0.05.

## 3. Results

### 3.1. HPLC and Fractionation of ADLE by Gradient CPC

**Figure 1:**
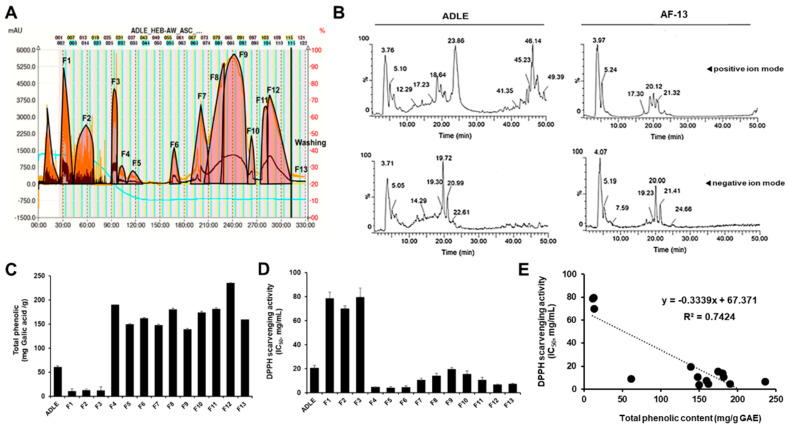
Correlation between total phenolic content and DPPH radical scavenging activity of fractions obtained after solvent fractionation of ADLE by gradient CPC. (**A**) Gradient CPC chromatogram of ADLE. (**B**) LC–MS chromatogram of ADLE and its fraction AF-13 in positive ion mode (top) and negative ion mode (bottom). (**C**) Total phenolic contents were expressed as mg of GAE/g of dry weight of fractions. (**D**) DPPH radical scavenging activity represented concentration of fractions required to inhibit 50% of the control calculated from linear regression equation. The final concentration of the DPPH radical was 98 µM. The concentration of each fraction was 1, 5, and 10 mg/mL. (**E**) Linear regression line between total phenolic content and DPPH radical scavenging activity of fractions. Coefficient of determination R^2^ = 0.74 (*p* < 0.05). Values are expressed as mean ± SD (*n* = 3).

Fractionation of ADLE was performed by a linear-gradient CPC using three ternary solvent systems. As shown in Figure 1A, 13 fractions were obtained, and the overall CPC operating time was 330 min. After CPC operation, the total quantity of each fraction was 4824.5 mg when 5.0 g of crude sample was loaded. The recovery rate was 96.5%. We also performed the LC–MS analysis of ADLE and the 13 subfractions were obtained. LC–MASS chromatogram of ADLE and AF-13 fraction are shown in Figure 1A,B. AF-13 is a part of the polar material eluted from the last washing step after CPC fractionation and corresponds to about 87.7% of ADLE.

### 3.2. Total Phenolic Contents and Antioxidant Activities of ADLE and Its Fractions

The total phenolic contents of the fractions were calculated from the regression equation of the calibration curve (*r*^2^ = 0.998, *y* = 1.05*x* − 0.006) and expressed in GAE as milligrams per gram of the sample (mg GAE/g sample). ADLE had 61.4 ± 3.4 mg GAE/g, and the total phenolic content of its 13 fractions ranged from 11.1 ± 3.4 to 236.0 ± 8.2 mg GAE/g sample. Free radical scavenging activity is one of the important factors for evaluating the antioxidant capacity derived from plants and other organisms [15]. Therefore, the dose-dependent antioxidant activity of the 13 fractions was determined by DPPH assay. As depicted in Figure 1C,D, five fractions (4,8,11,12,13) showed a relatively high polyphenol content as well as high antioxidant capacity. Figure 1E shows the correlation between phenolic contents (*x*-axis) and the antioxidant activities (IC_50_ values) (*y*-axis) of ADLE and its evaluated fractions. The equation describing such correlation is *y* = −0.334*x* + 67.371, and the coefficient of determination for evaluating the relationship is 0.74. Briefly, this result indicated that the phenolic content contributed to 74% of antiradical activities.

### 3.3. Compound AF-13 Attenuates Palmitate-Induced Cytotoxicity in INS-1 Cells

**Figure 2:**
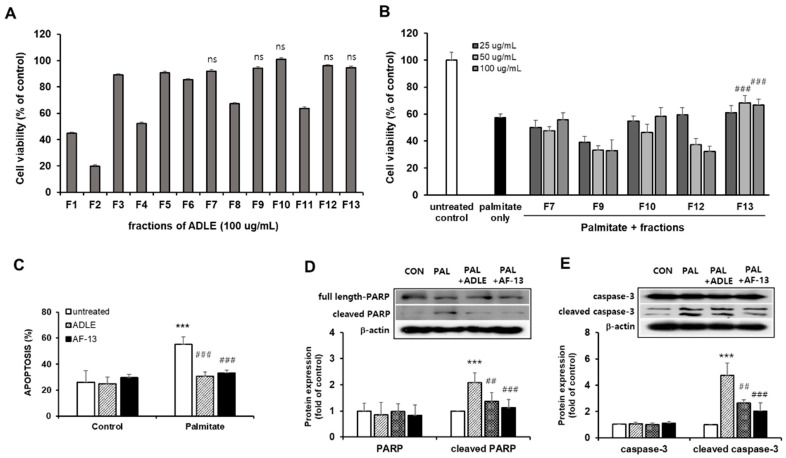
Effect of ADLE fractions on palmitate-induced cytotoxicity in INS-1 cells. (**A**) Effect of 13 fractions on the proliferation of INS-1 cells. Cells were incubated with fractions at 100 µg/mL for 24 h. (**B**) Palmitate (0.4 mM) was co-treated with the indicated concentration of fraction, and cell viability was determined at 24 h by MTT assay. (**C**) Quantification of apoptosis by fragmented DNA analysis using the Cell Death Detection ELISA kit. (**D**,**E**) Representative immunoblot and quantification of PARP and caspase-3. Data represent mean ± SD (*n* = 3–5). (Original western blot figure see Figure S1) CON: untreated control, PAL: 0.4mM palmitate-treated. Values are presented as mean ± SD from three independent experiments, normalized to the percentage of untreated control. *** *p* < 0.001 versus CON. ^##^ *p* < 0.01, and ^###^ *p* < 0.001 versus PAL. ns: no significance.

To examine whether the compounds have cytotoxicity, we investigated the viability of INS-1 cells after treating them with 100 µg/mL of compounds. We observed that treatment with eight compounds (F1–F6, F8, and F11) decreased the cell viability compared with the untreated control (Figure 2A). We next investigated whether five fractions (F7, F9, F10, F12, and F13), which have no cell toxicity, could protect INS-1 cells against palmitate-induced cell death. INS-1 cells were treated with various concentrations (25–100 µg/mL) of the compounds with or without 0.4 mM palmitate, which is known to induce beta cell death, for 24 h, after which the cell viability was measured. As shown in Figure 2B, cell viability was decreased in palmitate-treated cells compared with the control, and treatment with F13 (AF-13) (50–100 µg/mL) increased the cell viability (*p* < 0.001; Figure 2B). Treatment with F7, F9, F10, and F12 showed no increase in cell viability. Because treatment with AF-13 increased the viability of palmitate-treated cells, we compared this effect with ADLE treatment. In a preliminary study, we observed that treatment with 500 µg/mL of ADLE effectively suppressed palmitate-induced beta cell apoptosis (12). The occurrence of apoptosis and the levels of apoptosis-related proteins were evaluated in palmitate-treated cells in the absence or presence of the compound AF-13. The amount of nucleosomes in the cytoplasm upon DNA degradation was significantly increased due to palmitate toxicity, but there was a reduction after treatment with 100 µg/mL of AF-13 (Figure 2C, *p* < 0.001). Furthermore, the protein levels of cleaved PARP and caspase-3 induced by palmitate were significantly reduced by AF-13 treatment (Figure 2D,E). Similar results of apoptosis amount and its protein expression were observed with ADLE treatment (Figure 2D,E).

### 3.4. Compound AF-13 Prevents Nitrite, ROS, and TG Accumulation and Restores ATP Levels Reduced by Palmitate in INS-1 Cells

**Figure 3:**
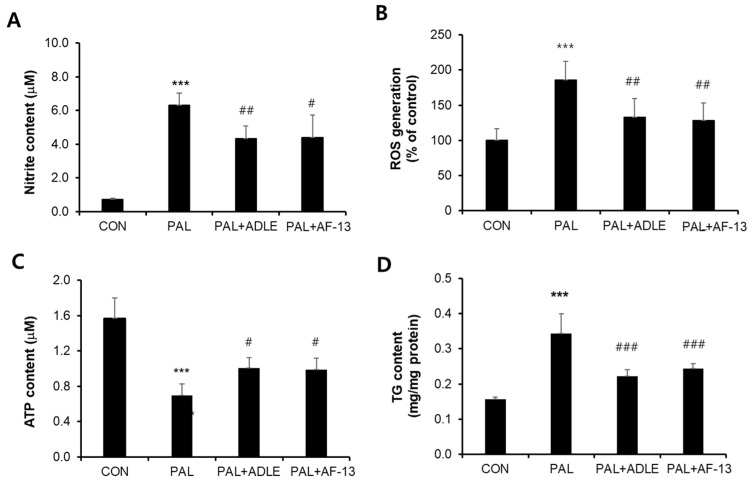
Effects of AF-13 on nitrite production (**A**), ROS generation (**B**), ATP content (**C**), and triglyceride (TG) content (**D**) in palmitate-induced INS-1 cells. Cells were treated with 0.4 mM PAL in the absence or presence of 500 µg/mL of ADLE and 100 µg/mL of AF-13 for 24 h. Data represent mean ± SD (*n* = 3–5). *** *p* < 0.001 versus CON, ^#^ *p* < 0.05, ^##^ *p* < 0.01, and ^###^ *p* < 0.001 versus PAL. CON: untreated control, PAL: 0.4 mM palmitate-treated.

Because AF-13 treatment reduced palmitate-induced beta cell death, we evaluated the effect of AF-13 treatment on palmitate-stimulated nitrite, ROS, and TG accumulation and ATP levels and also compared the effect with ADLE treatment [11]. Palmitate treatment of INS-1 cells for 24 h resulted in a 9-fold (*p* < 0.001) increase in nitrite accumulation compared with the control (CON). AF-13 treatment significantly decreased the nitrite levels to 33.9% (*p* < 0.01) compared with palmitate (PAL) treatment, and this effect was similar to that with ADLE treatment (Figure 3A). Intracellular ROS levels were detected by DCF fluorescein-labeled dye in palmitate-treated cells in the absence or presence of AF-13. The amount of fluorescence intensity of ROS was significantly increased by palmitate treatment (1.9-fold, *p* < 0.001 vs. CON), but there was a reduction after treatment with 100 µg/mL of AF-13 or 500 µg/mL of ADLE (Figure 3B). Furthermore, intracellular ATP levels were reduced by palmitate treatment compared with the control (56.3%, *p* < 0.001 vs. CON), but the levels were significantly improved by AF-13 treatment (18.8%, *p* < 0.05 vs. PAL) (Figure 3C). Palmitate treatment also induced the accumulation of approximately 2.1-fold higher levels of intracellular TG than those in CON, which were attenuated by AF-13 co-treatment (Figure 3D). Similar results were observed on ATP levels and TG content with ADLE treatment (Figure 3C,D).

### 3.5. Compound AF-13 Reduces Proinflammatory Factors through Inhibition of NF-κB Activation in Palmitate-Induced INS-1 Cells

**Figure 4:**
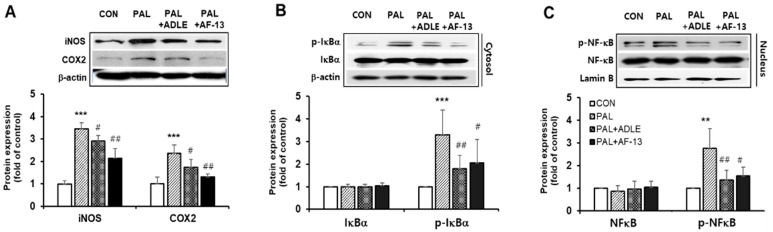
Effects of AF-13 on protein expression levels of inflammatory markers, and activation of NF-κB pathway in palmitate-induced INS-1 cells. (**A**) Representative immunoblot and quantification of iNOS and COX-2. (**B**,**C**) Representative immunoblot and quantification of NF-κB/IκB signal. (Original western blot figure see Figure S2) Data represent mean ± SD (*n* = 3–5). ** *p* < 0.01 and *** *p* < 0.001 versus CON, ^#^ *p* < 0.05 and ^##^ *p* < 0.01 versus PAL. CON: untreated control, PAL: 0.4mM palmitate-treated.

It has been reported that palmitate stimulates inducible nitric oxide synthase (iNOS) and cyclooxygenase-2 (COX-2) expression and that the upregulation of these proteins causes mitochondrial dysfunction in beta cells [16]. Therefore, we evaluated the effect of AF-13 treatment on palmitate-stimulated inflammation. Treatment with palmitate alone upregulated iNOS and COX-2 protein levels, whereas treatment with the compound AF-13 downregulated this effect (Figure 4A). We next examined whether AF-13 could inhibit the phosphorylation of IκB-α and translocation of NF-κB (active subunit p65) to the nucleus. Our results showed that cells treated with palmitate alone exhibited increased phosphorylation of IκB-α. However, palmitate-treated cells treated with AF-13 or ADLE exhibited a significant decrease in IκB-α phosphorylated protein levels (Figure 4B). Similar to the results obtained with IκB-α expression, palmitate treatment upregulated the protein levels of NF-κB p65 in the nuclear fraction, whereas the levels were significantly reduced by AF-13 or ADLE treatment (Figure 4C).

## 4. Discussion

Organic extracts derived from various sources are widely used in traditional medicine and food industry and are considered to be generally safe [10,17]. Among natural resources, insects have been focused for their effective bioactive products as a new class of anti-oxidant with potential clinical value for human beings [18]. We had previously reported that ADLE treatment ameliorated hepatic insulin resistance in high-fat diet-fed mice, enhanced intestinal permeability in LPS-treated Caco-2 cells, and reduced lipotoxicity in free fatty acid-induced pancreatic beta cells [11,12]. In the present study, to elucidate the bioactive compounds of ADLE, we isolated specific fractions and compared the effect on apoptosis and oxidative stress to that of ADLE.

In general, antioxidant activity shows good correlation with the content of GAE. Consistently, our data shown that DPPH activity was higher in AF-13 fraction. The results obtained from the in vitro system using DPPH scavenging might be reflect the in vivo results observed in high-fat-diet-induced diabetic mice [11].

Suh et al. reported that ADL extract exhibited an antioxidant effect by reducing the ROS scavenging activity and protected living organisms against photodamage [19]. They demonstrated that the presence of some phytochemicals, such as ascorbic acid, tocopherol, and pigments in ADLE is one of the major components that exhibits biological effects. Therefore, AF-13 could be a polar metabolite, such as a phytochemical compound or peptide containing multi-complex components.

Previously, we observed that ADLE treatment prevented apoptosis by reducing apoptotic signaling molecules such as cleaved caspase-3 and PARP in both INS-1 cells and isolated islets [12]. In this study, AF-13 treatment also increased the cell viability in palmitate-treated cells, which was primarily due to the reduction of apoptosis.

COX-2, also called prostaglandin-endoperoxide synthase-2, induces insulin resistance by synthesizing pro- and anti-inflammatory prostaglandins that can cause inflammation. COX-2 expression in most cells is known to be induced by pro-inflammatory cytokines and growth factors [20]. Amior et al. reported that palmitate upregulates the level of COX-2 expression in human islets and MIN6 pancreatic beta-cells and rises in islets isolated from type 2 diabetic patient [21]. We confirmed that palmitate increased COX-2 expression in INS-1 cells and that expression was more reduced in its active compounds AF-13 than in ADLE treatment.

In diabetic conditions, palmitate activates NF-κB, regulates the expression of proinflammatory genes, such as iNOS [22] and, consequently, disrupts the mitochondrial membrane potential [23]. Therefore, inhibition of NF-κB activation protects pancreatic beta cells against lipotoxicity-induced apoptosis. Treatment with AF-13 protected INS-1 cells against lipotoxicity by suppressing the NF-κB-dependent iNOS expression, thereby enhancing the levels of ATP.

Mitochondrial ROS is one of the major source of development of type 2 diabetes and long term treatment of palmitate in pancreatic beta cells, NOX-4 derived ROS generation was strongly increased [24,25]. In this study, we found that the generation of intracellular ROS levels by palmitate was significantly reduced in beta cells treated with AF-13. It is known that the cytosolic ROS was mainly originating in the mitochondria or producing by NOX-4, which localizes to mitochondria [26]. Moreover, ROS generated by palmitate in mitochondria may penetrate to the cytoplasm, increase cellular oxidative stress, and damage cellular components and DNA, which ultimately leads to cell death through apoptosis [27,28]. Therefore, the effect on mitochondrial ROS of AF-13 will show similar effects in obtained in the intracellular ROS. To determine whether anti-ROS effect of AF-13 was correlated with mitochondrial function, we measured cellular ATP content, which is one of the important indicators of mitochondrial function, in beta cells exposed to palmitate with or without AF-13. Relevant to the results of ROS generation, ATP content was significantly decreased lipotoxicity, and it was attenuated in cells treated with AF-13.

Nature bioactive compounds, such as quercetin, resveratrol, and curcumin modulates mitochondrial functions by altering production of mitochondria ROS and by modulating the expression of mitochondrial proteins [29,30]. Im et al. showed that antioxidant activity was increased by ADLE treatment in hairless mice skin exposed to ultraviolet B radiation [31], and we also confirmed the effect of ADLE and its active compounds AF-13 on palmitate-treated ROS production in pancreatic beta cells. These results suggested that AF-13 recovered mitochondrial function by reduced ROS generation, and these are one of the important factors regarding anti-apoptotic and anti-inflammatory effect of AF-13. Moreover, AF-13 possessing ROS scavenging ability could potentially prevent the risk of developing several free radical-mediated diseases [32].

Exposure to excess palmitate was found to induce ceramide accumulation, and *de novo* ceramide synthesis has been suggested as a mediator of free fatty acid-induced beta cell toxicity [33]. Ceramide increases mitochondrial membrane permeability and results in the activation of intrinsic pathways by decreasing the levels of anti-apoptotic molecules such as bcl-2 and increasing the levels of caspase-3 [34]. Our result showing the reduction of triglyceride content by AF-13 treatment is one of the mechanisms of anti-apoptosis.

The anti-apoptotic and anti-inflammatory effect of the compound AF-13 was higher than that of ADLE. We found that the effect observed by treatment with 100 µg/mL of AF-13 in palmitate-treated INS-1 cells was similar to the effect observed by treatment with 500 µg/mL of ADLE. These results suggested that AF-13 is one of the major compounds of ADLE responsible for biological activity in terms of the antioxidant effect.

The generation of ROS has been considered the ultimate cause of dysfunction in beta cells exposed to chronic FFA [35]. Particularly, beta cells have been reported susceptible to oxidative stress due to a high endogenous production of ROS and a low expression of antioxidative enzymes. ROS has been shown to activate NF-KB through alternative IkBa phosphorylation, which may results in the upregulation of target genes such as cytokines, pro-inflammatory genes and cox-2 [36,37,38]. Cox-2 that catalyses the formation of prostaglandin E2 is induced by interleukin-1; therefore, cox-2 is one of the important factors that contribute the development of diabetes and its complication [39]. These reports suggested that ROS/NF-kB/Cox-2 pathways plays an important role in the progression of inflammation and related disease including diabetes. Moreover, our results that reduction of these pathways by ADLE and AF-13 demonstrated that ADLE and AF-13 can be used to develop functional foods targeting diabetes.

In conclusion, our study findings suggest that AF-13, the major components of ADLE plays a vital role in ROS scavenging against oxidative stress. Furthermore, ethanol extraction could be a reliable method for obtaining ADE with acceptable yields of important compounds, although some further optimization of extraction procedures is still required.

## Data Availability

Not applicable.

## Supplementary Materials

The following are available online at https://www.mdpi.com/article/10.3390/life11060470/s1, Figure S1: Original scans of western blotdisplayed in Figure 2D,E. Figure S2: Original scan of western blot displayed in Figure 4A–C.
