# Supplementary material for: Anti-Inflammatory Activity of AF-13, an Antioxidant Compound Isolated from the Polar Fraction of Allomyrina dichotoma Larva, in Palmitate-Induced INS-1 Cells
Source: Life (Basel). 2021 May 24;11(6):470. doi: 10.3390/life11060470 (PMC8225099; doi:10.3390/life11060470)
Supplement: Supplementary file 1 [file life-11-00470-s001.zip › life-1210504-supplementary.pdf]

life

Article

Anti-Inflammatory Activity of AF-13, an Antioxidant Compound Isolated from the Polar Fraction of *Allomyrina Dichotoma* Larva, in Palmitate-Induced INS-1 Cells

Kyong Kim [1], Eun-Young Park [2], Dong-Jae Baek [2], Chul-Young Kim [3] and Yoon Sin Oh [1,*]

[1] Department of Food Nutrition, Eulji University, Seongnam 13135, Korea; kim_kyong@hanmail.net
[2] College of Pharmacy and Natural Medicine Research Institute, Mokpo National University, Jeonnam 58628, Korea; parkey@mokpo.ac.kr (E.-Y.P.); dbaek@mokpo.ac.kr (D.-J.B.)
[3] College of Pharmacy, Hanyang University, Ansan 15588, Korea; chulykim@hanyang.ac.kr
* Correspondence: ysoh@eulji.ac.kr

Abstract: This study was conducted to evaluate the fractions isolated from *Allomyrina dichotoma* larva extract (ADLE) that exhibited anti-apoptotic and anti-inflammatory effects. A total of 13 fractions were eluted from ADLE by centrifugal chromatography (CPC), and the polar AF-13 fraction was selected, which exerted a relatively protective effect against fat-induced toxicity in INS-1 cells. AF-13 treatment of palmitate-treated INS-1 cells decreased the expression level of apoptosis-related proteins and DNA fragmentation. AF-13 also significantly inhibited the production of nitric oxide and reactive oxygen species and the triglyceride content induced by palmitate, and the effect was found to be similar to that with ADLE treatment. Palmitate upregulated the expression of cyclooxygenase-2 (COX-2) and inducible nitric oxide synthase (iNOS) through the activation of NF-κB p65; however, this effect was significantly attenuated by AF-13 treatment. In conclusion, AF-13 is one of the major components of ADLE responsible for anti-apoptotic and anti-inflammatory activities.

Keywords: Allomyrina dichotoma larva; AF-13 fraction; palmitate; INS-1; inflammation; apoptosis

Citation: Kim, K.; Park, E.-Y.; Baek, D.-J.; Kim, C.-Y.; Oh, Y.S. Anti-Inflammatory Activity of AF-13, an Antioxidant Compound Isolated from the Polar Fraction of *Allomyrina Dichotoma* Larva, in Palmitate-Induced INS-1 Cells. *Life* **2021**, *11*, 470. https://doi.org/10.3390/life11060470

Academic Editor: Fabrizio Montecucco

Received: 20 April 2021
Accepted: 21 May 2021
Published: date

Publisher's Note: MDPI stays neutral with regard to jurisdictional claims in published maps and institutional affiliations.

Copyright: © 2021 by the authors. Submitted for possible open access publication under the terms and conditions of the Creative Commons Attribution (CC BY) license (http://creativecommons.org/licenses/by/4.0/).

Supplementary Materials:

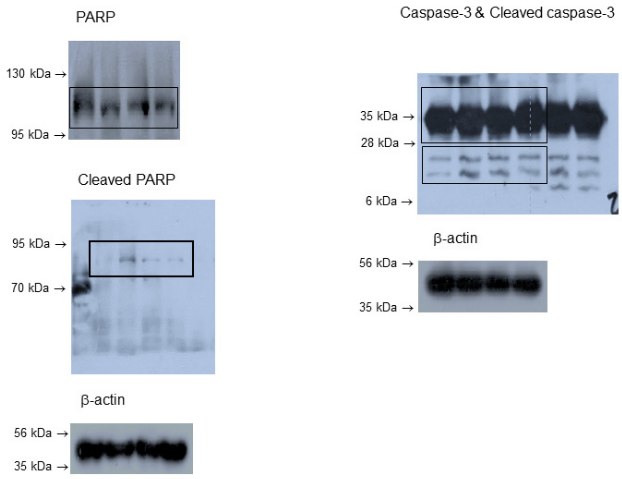

Figure S1. Original scans of western blotdisplayed in Figure 2D,E.

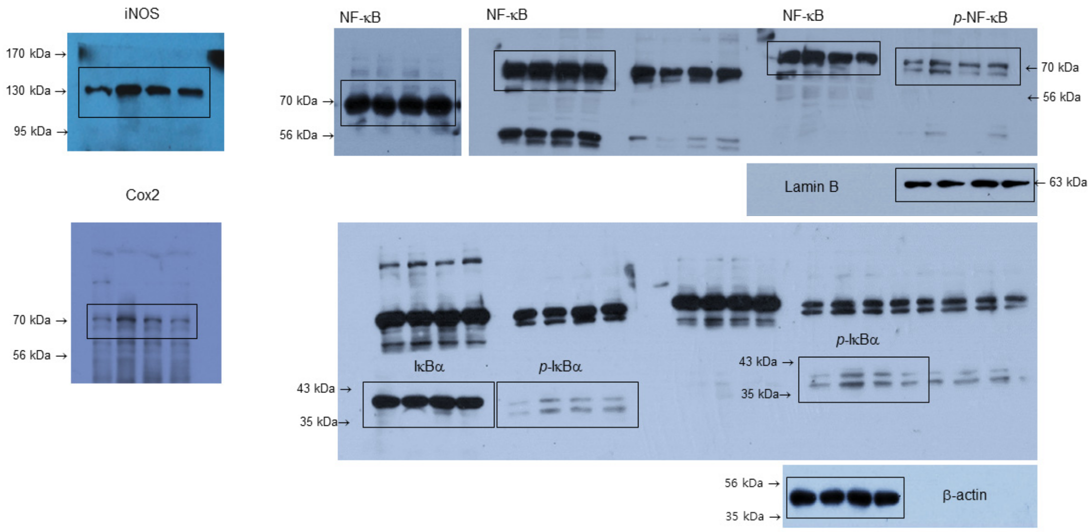

Figure S2. Original scan of western blot displayed in Figure 4 A–C.
